# Effects of aerobic exercise on lipids and lipoproteins

**DOI:** 10.1186/s12944-017-0515-5

**Published:** 2017-07-05

**Authors:** Yating Wang, Danyan Xu

**Affiliations:** Department of Cardiovascular Medicine, The Second Xiangya Hospital, Central South University, Changsha, Hunan 410011 China

**Keywords:** Aerobic exercise, Coronary heart disease, Dyslipidemia, Lipoprotein

## Abstract

Dyslipidemia is the risk of cardiovascular disease, and their relationship is clear. Lowering serum cholesterol can reduce the risk of coronary heart disease. At present, the main treatment is taking medicine, however, drug treatment has its limitations. Exercise not only has a positive effect on individuals with dyslipidemia, but can also help improve lipids profile. This review is intending to provide information on the effects of exercise training on both tranditional lipids, for example, low-density lipoprotein cholesterol, high-density lipoprotein cholesterol, triglycerides and new lipids and lipoproteins such as non-high-density lipoprotein cholesterol, and postprandial lipoprotein. The mechanisms of aerobic exercise on lipids and lipoproteins are also briefly described.

## Background

It has been consistently showed that concentration of low-density lipoprotein cholesterol (LDL-C) increasing is associated with an increased risk of myocardial infarction and vascular death [1]. High-density lipoprotein cholesterol (HDL-C) is a strong, consistent, and independent predictor of cardiovascular events, which has been confirmed by many prospective studies on different racial and ethnic groups worldwide [2, 3]. In addition, triglycerides (TG) can enter the arterial wall with a mild to moderate increase concentration (2–10 mmol/L), and then accumulate at there, thus causing the possibility of atherosclerosis [4]. Between 2007 and 2008, an increase in TG was associated with an increased risk of myocardial infarction, ischemic heart disease, ischemic stroke, and all-cause mortality, according to studies in the Copenhagen City Heart Study and Women’s Health Study [5–7].

Lowering serum cholesterol can help reduce the risk of coronary heart disease (CHD). Statin therapy has been appropriately emphasized in the current US and European guidelines as the primary treatment for LDL-C reduction because of strong evidence of reduced safety, efficacy and events [8, 9]. However, many people cannot tolerate statins, and statins are contraindicated in pregnant women. Therefore, there is a need to find another non-statin to help more people better reduce LDL-C. There are several novel methods of reducing LDL-C in active studies, such as inhibitors of mipomersen, lomitapide, and proproteinase/subtilisin/kexin 9 (PCSK9). However, their hepatotoxicity, high cost, inconvenience, and a general lack of availability outside the tertiary referral centers imply that their use is limited [10]. Researchers have also attempted to reduce cardiovascular risk by increasing HDL-C concentrations. There are two main approaches currently available: elevation of HDL-C directly, such as with cholesterol ester transfer protein (CETP) inhibitors; or promotion of the reverse cholesterol transport (RCT) pathway, e.g., infusion of apolipoprotein A-I (apoA-I) containing recombinant HDL particles or lipid-poor HDL particles [11]. However, there have been no positive outcomes. For the reduction of TG, more advice comes from changes in lifestyle, such as sugar and the Mediterranean diet [12, 13]. Of course, drugs are also effective [14], such as fibrates, fish oil and niacin [15].

## Aerobic exercise with lipids and lipoproteins

### Aerobic exercise

In addition to these treatments, aerobic exercise has been shown to improve the prognosis of cardiovascular disease (CVD). Aerobic exercise is defined as any form of physical activity that produces an increased heart rate and respiratory volume to meet the oxygen requirements of the activated muscle. Compared to medications, aerobic exercise is easier to carry out and has fewer side effects. Pedersen and Saltin [16] concluded that exercise can have a positive impact on symptoms and physical health via investigation of multiple meta-analyses about exercise and lipid profiles. Kokkinos et al. [17] performed a prospective cohort study of exercise and lipid metabolism. Individuals were grouped by evaluating the peak metabolic equivalents (MET) achieved during the exercise endurance test, the adaptation conditions and the different statin treatment. After 10 years, for individuals who took statins, the mortality risk decreased, while their fitness increased; the hazard ratio in patients who were in highly fit for highly fit (>9 MET) was 0.3 when compared with those who were in least fit (<5 MET). Therefore, the authors concluded that the risk of mortality is significantly reduced when combined with statin therapy and aerobic exercise compared to either method alone, and that aerobic exercise is required for individuals with dyslipidemia.

In their 2016 study, Ekelund et al. [18] investigated the effects of exercise and non-exercise on all-cause mortality. They conducted a prospective cohort study to analyze the effects of exercise versus non-exercise on cardiovascular mortality by analyzing the time of sitting and the duration of exercise. The study involved 1,005,791 individuals for a total follow-up of 2–18.1 years. Compared with the exercise group (i.e., > 35.5 MET-h per week), the mortality rate during follow-up was 12–59% higher for people who were sitting for a long time on a daily basis. In addition, they found that for people who exercised a lot every day, increasing their sitting time did not increase their all-cause mortality. As a result, the authors concluded that prolonged sitting could indeed increase all-cause mortality, but a certain amount of exercise per day seemed to counteract this effect to some extent.

For its low-cost, low-risk and non-drug intervention that can be applied to the vast majority of the public, aerobic exercise is recommended for CHD patients [19]. In 2015, the European Society of Cardiology recommends that aerobic exercise training in cardiac rehabilitation programs should provide aerobic exercise training in patients with non-ST-elevation acute coronary syndrome and the need to assess exercise capacity and exercise-related risks. If feasible, it is recommended that regular exercise training ≥3 three or more times a week and at 30-45 min per session [20].

### Aerobic exercise and HDL-C

Many studies have focused on the relationship between aerobic exercise and HDL-C, and have found that HDL-C levels are more sensitive to aerobic exercise than both LDL-C and TG. Furthermore, all studies focusing on the effects of exercise on HDL-C seemed to consistently indicate that there was an increase in HDL-C more or less, no matter in human or in rats (Tables 1 and 2). However, people usually improve their lifestyles as they do aerobic exercise, which can lead to interference with the results. To avoid this problem, Kodama et al. [28] performed a meta-analysis of 25 randomized controlled trials. People only exercised, that is, without medications or dietary therapy. Still, they found HDL-C increased by 2.53 mg/dL when aerobic exercise was 5.3 MET (64.8% maximal aerobic capacity).

**Table 1:** Several studies about effects of aerobic exercise on HDL-C, LDL-C and TG in human

References	n	Design	Training time	Training frequency	Training strength	Changes of HDL-C	Changes of LDL-C	Changes of TG
LeMura et al. [21]	12 women	RCT	16 weeks	3 sessions/week	70–85% of the HRmax	Increased 0.4 mmol/L	Decreased 0.2 mmol/L	Decreased 0.2 mmol/L
Nybo et al. [22]	36 men	RCT	12 weeks	150 min/week	65% VO2max	Increased 0.1 mmol/L	Decreased 0.1 mmol/L	Not mentioned
Kraus et al. [23]	111 men and women	RCT	24 weeks	Expand 14–23 kcal/kg/week	65–80% VO2max	Increased 4.3 mg/dL	Decreased 1.9 mg/dL	Decreased 28.4 mg/dL
O’Donovan et al. [24]	64 men	RCT	24 weeks	400 kcal/session 3 sessions/week	60% VO2max	Increased 0.08 mmol/L	Increased 0.17 mmol/L	Increased 0.12 mmol/L

**Table 2:** Several studies about effects of aerobic exercise on HDL-C, LDL-C and TG in rats

References	n	Design	Training time	Training frequency	Training strength	Changes of HDL-C	Changes of LDL-C	Changes of TG
Kazeminasab et al. [25]	12	RCT	8 weeks	5 times/week	20−28 m/min	0.461 mmol/L higher than control group	0.138 mmol/L lower than control group	0.292 mmol/L lower than control group
Ghanbari-Niaki et al. [26]	10	RCT	6 weeks	90 min/day 5 days/week	25 m/min	11.86 mg/dL higher than control group	1.02 mg/dL lower than control group	25.5 mg/dL lower than control group
Kazeminasab et al. [27]	12	RCT	4 weeks	1 h/day 5 days/week	20−28 m/min	4.67 mg/dL higher than control group	3.5 mg/dL lower than control group	1.33 mg/dL higher than control group

As there have been many studies on the relationship between aerobic exercise and HDL-C, researchers have begun to focus on the relationship between aerobic exercise and HDL-C subfractions. As evidence suggests that HDL2-cholesterol (HDL2-C) provides greater protection against coronary heart disease, researchers are paying more attention to HDL2-C than to HDL3-cholesterol (HDL3-C) [29]. However, there were not many significant positive results in previous randomized controlled trials, and only 30% of studies showed that aerobic exercise had an effect on HDL2-C [30, 31]. To determine the actual relationship between aerobic exercise and HDL2-C, Kelley et al. [32] studied the effect of aerobic exercise on adult HDL2-C using meta-analysis. They included 19 randomized controlled trials and found that HDL2-C increased by about 11% in subjects undergoing aerobic exercise, and the results were statistically significant. In addition, even if each study was removed from the model, the results still remained statistically significant. Besides, exercise-induced HDL2-C increase was not apparently associated with changes in body weight, body mass index, and body fat composition. HDL3-C is affected by aerobic exercise too. Halverstadt [33] conducted a study that included 100 healthy individuals who did not exercise previously. After 24 weeks of aerobic exercise, their HDL3-C levels were significantly reduced (1.9 ± 0.5 mg/dL, *p* = 0.01). In addition, studies have shown that, like HDL2-C, changes in plasma HDL3-C induced by aerobic exercise training were independent of changes in diet and body fat.

The main function of HDL-C is to participant the RCT process. Králová Lesná et al. [34] found that exercise could increase cholesterol efflux by 1.8% after 9 weeks training. Besides RCT, HDL-C possesses other functions such as clear lipid peroxide transport. Välimäki IA et al. [35] and Tiainen et al. [36] found that exercise could increase oxidized HDL (oxHDL) and oxHDL/HDL and decrease oxLDL at the same time. So, exercise can affect the HDL-C function including RCT process and lipid peroxide transport clearing. And the function of anti-inflammation or anti-oxidation needs further research.

### Aerobic exercise and LDL-C

Fasting LDL-C is strongly associated with an increased risk of coronary artery disease (CAD), so it is necessary to make clear the effect of aerobic exercise on LDL-C. Unlike HDL-C, the effect of exercise on LDL-C is inconsistent in human and there are even completely contrary results (Table 1). The results of these different studies may be due to variations in people’s weight. Some studies showed that aerobic exercise alone did not change the fasting blood LDL-C levels, unless the weight during this period also changed. In addition, research statistics showed that per kilogram of body weight loss resulting in LDL-C reduced by about 0.8 mg/dL [37].

In rats, the changes of HDL-C are almost the most obviously one than others. It may not only because HDL-C is more vulnerable to exercise, but also because HDL-C is the most abundant of lipids component in rats. Besides, different from the human being, LDL-C consistently decreased after exercise in rats (Table 2).

Although the current results on the LDL-C response to the aerobic exercise are discordant, studies have still indicated the potential occurrence of important cardioprotective improvements in LDL-C subfractions. LDL-C is classified according to their size and density. LDL-C subfractions that directly related to cardiovascular events are smaller, denser LDL particles [38]. Because of the knowledge of different LDL subcomponents, it has become necessary and interesting to explore whether aerobic exercise particularly affects certain LDL subcomponents. In some patients with mild to moderate dyslipidemia, the researchers found that after a few months of aerobic exercise, LDL-C did not change significantly, but the concentration of atherogenic small LDL particles decreased, and the average size of LDL particles increased [39]. Therefore, the impact of aerobic exercise on LDL-C should not be limited to total LDL-C, but LDL-C subfractions should also be considered. However, Varady et al. [40] found that the LDL particle volume decreased in patients with hypercholesterolemia after aerobic exercise. Therefore, they worry that aerobic exercise may reduce the LDL particle volume to increase CHD risk. By contrast, Elosua et al. [40] suggested that aerobic exercise had no effect on LDL particle diameter. Given these discrepant results, additional studies addressing the effects of aerobic exercise on the LDL fraction appear to be necessary.

Plasma Lp (a) is another LDL subunit containing Apo (a). However, unlike other low-density lipoprotein subfractions, Lp (a) is genetically influenced, unaffected by motion, and cannot be improved by any form of exercise [41].

ApoB is a major component of LDL particles and is essential for the removal of LDL particles in the circulation. About 95% of apoB binds to LDL particles, and each LDL particle binds to only one apoB molecule [42]. Therefore, the concentration of apoB indirectly reflects the concentration of LDL-C to a certain extent. Because of this, increased apoB concentrations can reflect an increased cardiovascular risk [43]. The effects of aerobic exercise on apoB concentration are not well confirmed. Crouse et al. [44] and Laaksonen et al. [45] both found that after a few months of aerobic exercise, the concentration of apoB in hypercholesterolemic men decreased. However, there were controversies about these findings. For example, Leon et al. [46] found that 20 weeks of aerobic exercise did not affect the concentration of apoB. Others found no change in apoB concentrations during either long (48 weeks) or short (3 weeks) aerobic exercise. Some factors must result in these various outcomes, for example, age. Accordingly, the authors designed a study to determine whether age influenced the results, however, they found that age did not affect apoB or lipoprotein concentrations in response to exercise [47]. Therefore, more studies on aerobic exercise and apoB remain necessary.

### Aerobic exercise and TG

It is widely reported that exercise can induce lower plasma TG concentrations (Tables 1 and 2). However, many studies have shown that sedentary individuals have no change in TG levels after a single exercise session [48]. The reasons for the discrepancies were unclear. It seemed that where there was an exercise with a high energy requirement, there would be more frequent that TG concentrations decreases occur. Actually, TG changes in sedentary subjects did occur regardless of whether the energy consumption is low or moderate [49]. Therefore, the energy consumption may not be the main reason for this distinction. If this is the case, is it the state of the patient before exercise (e.g., sitting or exercise) is the key factor? To our disappointment, the TG did not change significantly in non-active subjects with aerobic exercise. But the results of other studies have been very gratifying. The researchers found that when participants had lower baseline levels of TG, there was only a slight decrease in TG after exercise. While, when TG baseline levels were high, there was a significant reduction. Thus, the TG baseline level may be the key factor influencing the effect of exercise on the TG response.

It has been approved that the baseline level of TG are controversy to that of HDL-C, HDL2-C, and HDL3-C. However, investigators have noted the HDL-C increasing but not TG level decreasing at the same time in subjects after an aerobic exercise training [50]. Numerous studies showed that TG reduction always accompanied with no vary of HDL-C, or HDL-C increased with no apparent improvements of TG level. Likewise, in previous inactive individuals, HDL-C elevation has no relationship with TG level. But, it has been approved that there were both HDL-C increasing and TG decreasing with sedentary hyperlipidemia participants after aerobic exercise training. For instance, Peter et al. noted that after 2 days at the end of the aerobic exercise, the TG decreased with an HDL-C increase approximated to 14%. HDL-C elevation closing to 11% may contribute to the HDL-C concentrations raise [51]. At present, researchers are puzzled the different responses of TG and HDL-C to the aerobic exercise training. It appears that body weight, body fat, cardiovascular fitness, training status, regional lipid concentration, dietary changes, and genetic factors all contribute to it. In addition, exercise intensity, exercise time, as well as blood collection time, blood test technology, subject sample size should also be taken into account.

### Aerobic exercise and postprandial lipemia

There are a number of studies that provide important evidence for exercise training in response to lipids. However, almost all of the blood collection is in fasting state. Therefore, it can only reflect the effect of exercise on fasting lipid. As we all know, only a few hours before breakfast can be strictly referred to as fasting state, and the state of time after a meal is far more than fasting state. Therefore, the researchers speculated that postprandial lipid was of more sense in lipid metabolism than fasting state, and postprandial lipid may have a greater role than fasting blood lipids in the prediction of cardiovascular risk factors. In addition, the speculation has been approved by finding that postprandial triacylglycerolemia predicts cardiovascular events better than fasting triacylglycerol (TAG) concentrations [6]. Mestek et al. [52] observed that exercise training reduced the non-fasting TG response to a high-fat diet in individuals with metabolic syndrome. Postprandial blood lipid response to aerobic exercise not only occurred immediately after training in the acute phase, but also lasted to the next day. In addition, there is no need for a specific exercise. Exercise can be done all day. There was no significant difference between continuous aerobic exercise and single-session effectiveness in reducing non-fasting TG levels [53]. Sabaka et al. [54] found that exercise for 4 days resulted in significantly changes of postprandial TG, LDL-C and VLDL remnants. However, there was no significant change in postprandial HDL-C, but some HDL-C subfractions were altered. For example, the authors noted a statistically significant reduction in small and medium HDL particles in the postprandial state after exercise training. Therefore, aerobic exercise does affect postprandial lipid distribution. Another important finding was that only 4 days of physical exercise can lead to significant positive changes in postprandial lipid profile, suggesting that short-term aerobic exercise can improve postprandial lipid distribution.

Vigorous exercise training can significantly reduce postprandial lipids, which is more common in healthy and non-obese individuals. To investigate the effects of vigorous exercise on postprandial TG, subjects exercised before a high fat diet, but exercised for longer periods of time and exercised more intensively [55, 56]. As respected, there was substantial lowering of postprandial TG level. However, for most people, especially cardiovascular patients, such a high intensity of exercise is not appropriate. In fact, this intensive training is not necessary. In order to reduce postprandial blood lipids, moderate-intensity aerobic exercise is sufficient. In addition, low-fitness people only need to use low-intensity aerobic exercise [57]. Therefore, despite the high-intensity aerobic exercise have a very significant impact on postprandial lipid changes, for most people, moderate or lower intensity exercise is sufficient.

### Aerobic exercise and non–HDL-C

Recent evidence suggested that non-HDL-C was a better indicator of CVD risk than traditional lipids such as HDL-C, LDL-C, and TG. In addition, studies have shown that, as a predictor of future cardiovascular risk, non-HDL-C was more persuasive than LDL-C to some extent. Given that the potential benefits of aerobic exercise and the risk associated with elevated non–HDL-C levels, meta-analysis has been used to examine the effects of aerobic exercise on non–HDL-C in children and adolescents. However, previous meta-analysis reported that walking reduced adult non-HDL-C by 4% [58]. In contrast, one study reported that non-HDL-C in the exercise training group did not change significantly compared with the control group. The subjects were children and adolescent whose TC and HDL-C levels were essentially normal, and both of which were critical factors in the calculation of non-HDL-C, thus did not achieve the desired positive results. However, few studies have focused on the relationship between aerobic exercise and non-HDL-C levels. Accordingly, more studies should be planned to illustrate the relationship between the two.

### Related influencing factors of aerobic exercise on lipids

Many factors lead to different results on the effects of aerobic exercise on lipoprotein levels, such as various training time or training intensities. Some researchers believe that, to keep this effect longer, then, aerobic exercise time also needs longer, as well as the intensity needs more intensive. Dunn et al. [59] suggested that short-term training could also make improvements on plasma lipids, as long as there was enough exercise intensities. Kraus et al. [23] observed that the total energy consumption and exercise intensity was the main factor affecting lipid changes. O’Donovan et al. [24] aimed to study the effect of exercise intensity on lipid changes and found that, in the same amount of exercise, the exercise intensity is higher, the more obvious changes in blood lipids.

The results of these studies suggest that exercise time, exercise volume and exercise intensity all have an effect on exercise-induced changes in blood lipids. HDL-C is the most sensitive to exercise. In order to reduce LDL-C and TG levels more, it is necessary to increase the aerobic exercise intensity. However, this is difficult to achieve in individuals with coronary artery disease who are of limited exercise capacity or other risk factors.

### The mechanisms of the effects of aerobic exercise on lipids

Although the mechanism of exercise-induced lipid changes is unclear, exercise itself may increase blood lipid consumption hence to decrease lipids levels [60]. Mechanisms may involve the increased activity of lipoprotein lipase (LPL) - lipoprotein lipase responsible for chylomicrons and VLDL TAG hydrolysis in granules [61]. Most of the catalytically active LPL is located in the vessel wall and then isolated from the endothelium surface and released in the blood after intravenous injection of heparin [62]. Therefore, the detected LPL is often the post-heparin LPL. Ferguson et al. [63] reported that heavy or prolonged aerobic exercise episodes could significantly increase post-heparin plasma LPL activity, thus promoted LPL-mediated TG hydrolysis. However, with several findings showing that pre-heparin LPL concentration indicate the amount of systemic LPL activity [64–66], Tanaka et al. turned the target to pre-heparin LPL, and found that 12 weeks of jogging training increased pre-heparin LPL concentrations in overweight men [67]. Exercise-induced LPL changes were time-delayed, for example, LPL mRNA peak level occurred at 4 h after exercise [62]. Besides, LPL activation elevation could last for 24 h after only a 1 h exercise session in individuals with moderate intensity exercise [68].

In addition to the traditional mechanisms described above, several other discoveries revealed the mechanisms about exercise altering lipids profile from other aspects. Increased expression of ATP-binding cassette transporter A-1 (ABCA1) in macrophages has a strong effect on RCT, plasma HDL-C formation, and protection against atherosclerosis. So far, studies focused on the impact of aerobic exercise on blood ABCA1. Study found that the ABCA1 gene expression was significantly different before and after exercise [69]. Ghanbari-Niaki et al. [70] also found that ABCA1 mRNA expression increased regardless of the intensity of exercise. Therefore, they hypothesized that aerobic exercise may increase the expression of ABCA1 to exert its role in reducing cardiovascular risk. The researchers tested this hypothesis in a recent study [26]. They used human CETP transgenic (CETP-tg) mice to study the effects of aerobic exercise on RCT. Male CETP-tg mice were randomly assigned to control and exercise groups. Six weeks later, they found ABCA1 protein levels increased 100% in the liver of the exercise group.

Liver X receptor (LXR) is one of the transcription factors of nuclear receptor superfamily that play a key role in liver cholesterol metabolism. A study reported that low intensity exercise resulted in significant increase in LXR expression in human [71]. Study showed that LXRα expression was significantly elevated 2.8 fold in exercised rats than the control group [27]. LXR has been proved involving in regulating the expression of ABCA1. So, exercise may by inducing higher LXR and ABCA1 to improve the RCT process, which resulting in increased plasma HDL-C levels.

PCSK9 is a hot spot in the field of cardiovascular research in recent years as it is a new biomarker of LDL clearance and a new target of CVD therapy. Exercise can reduce plasma LDL-C levels, and PCSK9 plays an important role on the regulation of LDL receptor. Therefore, the investigators have considered that exercise is likely to affect LDL-C by modulating PCSK9. Kamani et al. [72] found a significant decrease in mean PCSK9 levels and mean LDL-C levels in volunteers after 3 months exercise, and concluded that daily exercise is independently associated with a decrease in PCSK9 levels over time. Rideout et al. [73] used C57BL/6 mice as a model, and feed them with high-fat diet, then make them do aerobic exercise. After 8 weeks, the levels of PCSK9 mRNA and sterol regulatory element binding protein 2 (SREBP2) were increased significantly in mice with high-fat diet and exercise training, 1.9 and 1.8 times higher than those only with high-fat diet respectively. In addition, both plasma PCSK9 and cholesterol were reduced by 14% in mice with both high-fat diet and exercise training, while no change with those only with high-fat diet. Accordingly, one means by which aerobic exercise helps improve lipoprotein levels may be via PCSK9 or SREBP2. However, additional mechanistic studies are required to directly link exercise-induced lipid lowering with reduced PCSK9 activity.

**Fig. 1:**
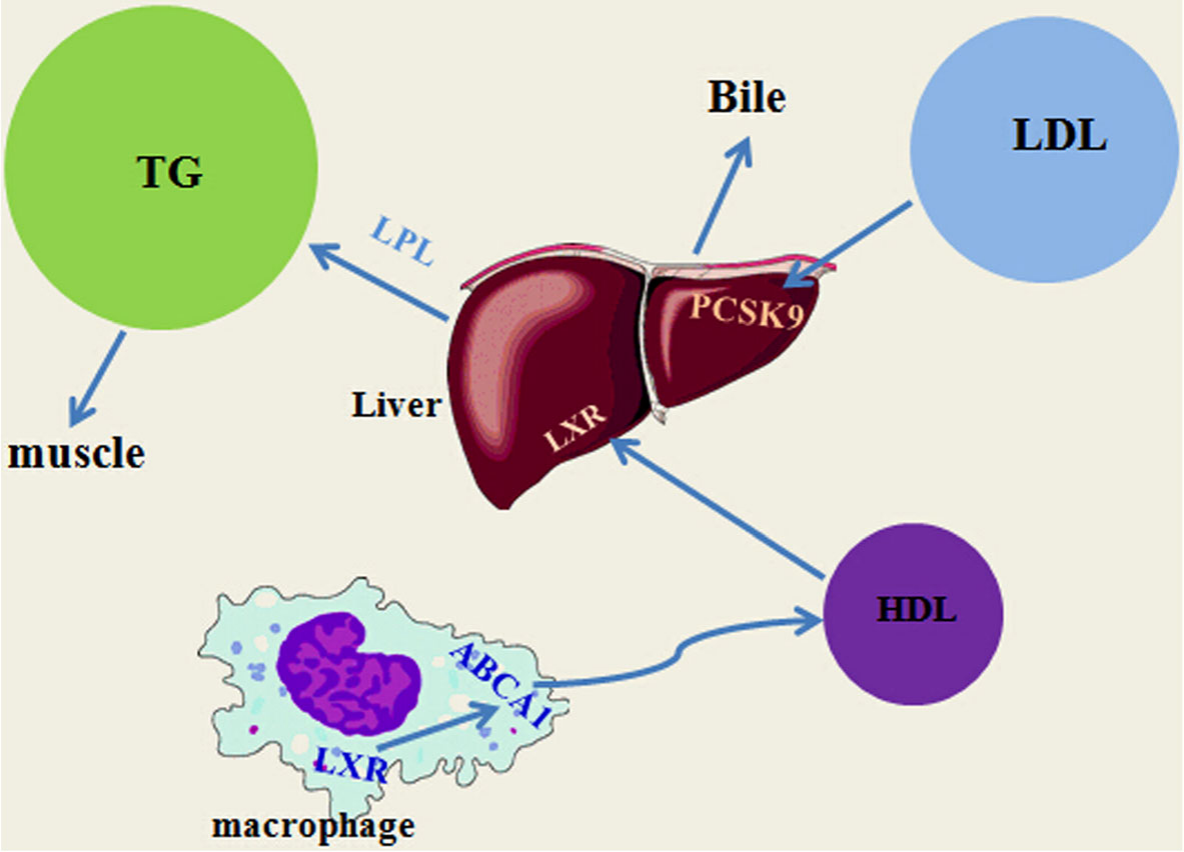
Summary of the potential mechanisms through which exercise improves the lipid profile. Legend: Exercise leads TG consumed by muscle tissue and increases LPL which results in more TG hydrolysis. Less PCSK9 makes more LDL absorbed and excreted by the liver. Upregulation of LXR increased ABCA1 expression in macrophage and then promoted RCT process, which results more cholesterol transported to the liver via HDL

Summary of the potential mechanisms through which exercise improves the lipid profile is in Fig. 1.

## Conclusion

Currently, clinicians may be excessively reliant on lipid-lowering drugs (i.e., statins) to treat patients with dyslipidemias. In our opinion, aggressive lifestyle alterations, such as exercise, should not be abandoned. Such knowledge should aid in preventing and treating dyslipidemia while reducing the risks of myocardial infarctions and CAD. Clinicians should encourage as much physical activity as possible.

## Abbreviations

ABCA1: ATP-binding cassette transporter A-1
ApoA-I: Apolipoprotein A-I
CAD: Coronary artery disease
CETP: Cholesterol ester transfer protein
CHD: Coronary heart disease
CVD: Cardiovascular disease
HDL2-C: HDL2-cholesterol
HDL3-C: HDL3-cholesterol
HDL-C: High-density lipoprotein cholesterol
LCAT: Lecithin-cholesterol acyltransferase
LDL-C: Low-density lipoprotein cholesterol
LPL: Lipoprotein lipase
MET: Metabolic equivalents
PCSK9: Proprotein convertase subtilisin kexin 9
RCT: Reverse cholesterol transport
SREBP2: Sterol regulatory element binding protein 2
TAG: Triacylglycerol
TG: Triglycerides

## References

[1] Lewington S, Whitlock G, Clarke R (2007). Blood cholesterol and vascular mortality by age, sex, and blood pressure: a meta-analysis of individual data from 61 prospective studies with 55000 vascular deaths. Lancet.

[2] Toth PP, Barter PJ, Rosenson RS (2013). High-densith lipoproteins: a consensus statement from the National Lipid Association. J Clin Lipidol.

[3] Goff DC, Lloyd-Jones DM, Bennett G (2014). 2013 ACC/AHA guideline on the assessment of cardiovascular risk: a report of the American College of Cardiology/American Heart Association Task Force on Practice Guidelines. Circulation.

[4] Nordestgaard BG, Wootton R, Lewis B (1995). Selective retention of VLDL, ODL, and LDL in the arterial intima of genetically hyperlipidemic rabbits in vivo. Molecular size as a determinant of fractional loss from the intima-inner media. Arterioscler Thromb Vasc Biol.

[5] Nordestgaard BG, Benn M, Schnohr P, Tybjaerg-Hansen A (2007). Nonfasting triglycerides and risk of myocardial infarction, ischemic heart disease, and death in men and women. JAMA.

[6] Bansal S, Buring JE, Rifai N, Mora S, Sacks FM, Ridker PM (2007). Fasting compared with nonfasting triglyceride and risk of cardiovascular events in women. JAMA.

[7] Freiberg JJ, Tybjaerg-Hansen A, Jensen JS, Nordestgaard BG (2008). Nonfasting triglycerides and risk of ischemic stroke in the general population. JAMA.

[8] Baigent C, Blackwell L, Emberson J (2010). Efficacy and safety of more intensive lowering of LDL cholesterol: a meta-analysis of data from 170 000 participants in 26 randomised trials. Lancet.

[9] Mihaylova B, Emberson J, Blackwell L (2012). The effects of lowering LDL cholesterol with statin therapy in people at low risk of vascular disease: meta-analysis of individual data from 27 randomised trial. Lancet.

[10] Ridker PM (2014). LDL cholesterol: controversies and future therapeutic directions. Lancet.

[11] Rader DJ, Kees Hovingh G (2014). HDL and cardiovascular disease. Lancet.

[12] Hegele RA, Ginsberg HN, Chapman MJ (2014). The polygenic nature of hypertriglyceridaemia: implications for definition, diagnosis, and management. Lancet Diabetes Endocrinol.

[13] Berglund L, Brunzell JD, Goldberg AC (2012). Evaluation and treatment of hypertriglyceridemia: an Endocrine Society clinical practice guideline. J Clin Endocrinol Metab.

[14] Reiner Z, Catapano AL, De Backer G (2011). ESC/EAS guidelines for the management of dyslipidaemias: the Task for the management of dyslipidaemias of the European Society of Cardiology (ESC) and the European atherosclerosis Society (EAS). Eur Heart J.

[15] Nordestgaard BG, Varbo A (2014). Triglycerides and cardiovascular disease. Lancet.

[16] Pedersen B, Saltin B (2006). Evidence for prescribing exercise as therapy in chronic disease. Scand J Med Sci Sports.

[17] Kokkinos PF, Faselis C, Myers J (2013). Interactive effects of fitness and statin treatment on mortality risk in veterans with dyslipidaemia: a cohort study. Lancet.

[18] Eklund U, Steene-Johannessen J, Broun WJ (2016). Does physical activity attenuate, or even eliminate, the detrimental association of sitting time with mortality? A harmonized meta-analysis of data from more than 1 million men and women. Lancet.

[19] National Cholesterol Education Program, National Heart Lung and Blood Institute, National Institutes of Health (2002). Third report of the National Cholesterol Education Program (NCEP) expert panel on detection, evaluation, and treatment of high blood cholesterol in adults (adult treatment panel III) final report. Circulation.

[20] Roffi M, Patrono C, Collet JP (2016). 2015 ESC guidelines for the management of acute coronary syndromes in patients presenting without persistent ST-segment elevation: Task Force for the Management of Acute Coronary Syndromes in patients presenting without persistent ST-segment elevation of the European Society of Cardiology (ESC). Eur Heart J.

[21] LeMura L, von Duvillard S, Andreacci J (2000). Lipid and lipoprotein profiles, cadiovascular fitness, body composition, and diet during and after resistance, aerobic and combination training in young women. Eur J Appl Physiol.

[22] Nybo L, Sundstrup E, Jakobsen M (2010). High-intensity training versus traditional exercise interventions for promoting health. Med Sci Sports Exerc.

[23] Kraus W, Houmard J, Duscha B (2002). Effects of the amount and intensity of exercise on plasma lipoproteins. N Engl J Med.

[24] O’Donovan G, Owen A, Bird S (2005). Changes in cardiorespiratory fitness and coronary heart disease risk factors following 24 wk of moderate- or high-intensity exercise of equal energy cost. J Appl Physiol.

[25] Kazeminasab F, Marandi M, Ghaedi K (2013). Endurance training enhances LXRα gene expression in Wistar male rats. Eur J Appl Physiol.

[26] Ghanbari-Niaki A, Khabazian BM, Hossaini-Kakhak SA (2007). Treadmill exercise enhances ABCA1 expression in rat liver. Biochem Biophys Res Commun.

[27] Kazeminasab F, Marandi M, Ghaedi K (2017). Effects of a 4-week aerobic exercise on lipid profile and expression of LXRα in rat liver. Cell J.

[28] Kodama S, Tanaka S, Saito K (2007). Effect of aerobic exercise training on serum levels of high-density lipoprotein cholesterol: a meta-analysis. Arcb Intern Med.

[29] Morgan J, Carey C, Lincoff A (2004). High-density lipoprotein subfractions and risk of coronary artery disease. Curr Atheroscler Rep.

[30] Ballentyne FC, Clark RS, Simpson HS (1982). The effect of moderate physical exercise on the plasma lipoprotein subfractions of male survivors of myocardial infarction. Circulation.

[31] Wood PD, Stefanick MK, Dreon DM (1988). Changes in plasma lipids and lipoproteins in overweight men during weight loss through dieting as compared with exercise. N Engl J Med.

[32] Kelley GA, Kelley KS (2006). Aerobic exercise and HDL2-C: a meta-analysis of randomized controlled trials. Atherosclerosis.

[33] Halverstadt A, Phares DA, Wilund KR (2007). Endurance exercise training raises high-density lipoprotein cholesterol and lowers small low-density lipoprotein and very low-density lipoprotein independent of body fat phenotypes in older men and women. Metabolism.

[34] Králová Lesná I, Suchánek P, Kovár J (2009). Life style change and reverse cholesterol transport in obese women. Physiol Res.

[35] Välimäki IA, Vuorimaa T, Ahotupa M (2016). Strenuous physical exercise accelerates the lipid peroxide clearing transport by HDL. Eur J Appl Physiol.

[36] Tiainen S, Luoto R, Ahotupa M (2016). 6-mo aerobic exercise intervention enhances the lipid peroxide transport function of HDL. Free Radic Res.

[37] Goldberg AC, Hopkins PN, Toch PP (2011). Familial hypercholesterolemia: screening, diagnosis and management of pediatric and adult patients: clinical guidance from the National Lipid Association Expert Panel on familial hypercholesterolemia. J Clin Lipidol.

[38] Davidson MH, Ballantyne CM, Jacobson TA (2011). Clinical utility of inflammatory markers and advanced lipoprotein testing: advice from an expert panel of lipid specialists. J Clin Lipidol.

[39] Varady KA, St-Pierre AC, Lamarche B, Jones PJ (2005). Effect of plant sterols and endurance training on LDL particle size and distribution in previously sedentary hypercholesterolemic adults. Eur J Clin Nutr.

[40] Elosua R, Molina L, Fito M (2003). Response of oxidative stress biomarkers to a 16-week aerobic physical activity program, and to acute physical activity, in healthy young men and women. Atherosclerosis.

[41] Durstine JL, Grandjean PW, Cox CA, Thompson PD (2002). Lipids, lipoproteins, and exercise. J Cardpulm Rehabil.

[42] Shelness GS, Sellers JA (2001). Very-low-density lipoprotein assembly and secretion. Curr Opin Lipidol.

[43] Lamarche B, Moorjani S, Lupien PJ (1996). Apolipoprotein A-I and B levels and the risk of ischemic heart disease during a five-year follow-up of men in the Quebec cardiovascular study. Circulation.

[44] Crouse SF, O’Brien BC, Grandjean PW (1997). Effects of training and a single session of exercise on lipids and apolipoproteins in hypercholesterolemic men. J Appl Physiol.

[45] Laaksonen DE, Atalay M, Niskanen LK (2000). Aerobic exercise and the lipid profile in type 1 diabetic men: a randomized controlled trial. Med Sci Sports Exerc.

[46] Leon AS, Rice T, Mandel S (2000). Blood lipid response to 20 weeks of supervised exercise in a large biracial population: the HERITAGE family study. Metabolism.

[47] Angelopoulos TJ, Sivo SA, Kyriazis GA (2007). Do age and baseline LDL cholesterol levels determine the effect of regular exercise on plasma lipoprotein cholesterol and apolipoprotein B levels?. Eur J Appl Physiol.

[48] Kantor M, Cullinane E, Sady S (1984). Exercise acutely increases high density lipoprotein-cholesterol and lipoprotein lipase activity in trained and untrained men. Metabolism.

[49] Crouse SF, O’Brien B, Rohack JJ (1995). Changes in serum lipids and apolipoproteins after exercise in men with high cholesterol: influence of intensity. J Appl Physiol.

[50] Dufaux B, Order U, Muller R (1986). Delayed effects of prolonged exercise on serum lipoproteins. Metabolism.

[51] Grandjean PW, Crouse SF, Rohack JJ (2000). Influence of cholesterol status on blood lipid and lipoprotein enzyme responses to aerobic exercise. J Appl Physiol.

[52] Mestet ML, Plaisance EP, Ratcliff LA (2008). Aerobic exercise and postprandial lipemia in men with the metabolic syndrome. Med Sci Sports Exerc.

[53] Li J, Siegrist J (2012). Physical activity and risk of cardiovascular disease: a meta-analysis of prospective cohort studies. Int J Environ Res Public Health.

[54] Sabaka P, Kruzliak P, Balaz D (2015). Effect of short term aerobic exercise on fasting and postprandial lipoprotein subfractions in healthy sedentary men. Lipids Health Dis.

[55] Gill JM, Al-Mamari A, Ferrell WR (2004). Effects of prior moderate exercise on postprandial metabolism and vascular function in lean and centrally obese men. J Am Coll Cardiol.

[56] Peddie MC, Rehrer NJ, Perry TL (2012). Physical activity and postprandial lipidemia: are energy expenditure and lipoprotein lipase activity the real modulators of the positive effect?. Prog Lipid Res.

[57] Dekker MJ, Graham TE, Ool T (2010). Exercise prior to fat ingestion lowers fasting and postprandial VLDL and decreases adipose tissue IL-6 and GIP receptor mRNA in hypertriacylglycerolemic men. J Nutr Biochem.

[58] Kelley GA, Kelley KS, Tran ZV (2005). Walking and non-HDL-C in adults: a meta-analysis of randomized controlled trials. Prev Cardiol.

[59] Dunn A, Marcus B, Kampert J (1997). Reduction in cardiovascular disease risk factors: 6-month results from project active. Prev Med.

[60] Earnest CP, Artero EG, Sui X (2013). Maximal estimated cardiorespiratory fitness, cardiometabolic risk factors, and metabolic syndrome in the aerobics center longitudinal study. Mayo Clin Proc.

[61] Calabresi L, Franceschini G (2010). Lecithin: cholesterol acyltransferase, high-density lipoproteins, and atheroprotection in humans. Trends Cardiovasc Med.

[62] Miyashita M, Eto M, Sasai H (2010). Twelve-week jogging training increases pre-heparin serum lipoprotein lipase concentrations in overweight/obese middle-aged men. J Atheroscler Thromb.

[63] Kobayashi J, Nohara A, Kawashiri M (2007). Serum lipoprotein lipase mass: clinical significance of its measurement. Clin Chim Acta.

[64] Ferguson MA, Alderson NL, Trost SG (1998). Effects of four different single exercise sessions on lipids, lipoproteins, and lipoprotein lipase. J Appl Physiol.

[65] Hirano T, Nishioka F, Murakami T (2004). Measurement of the serum lipoprotein lipase concentration is useful for studying triglyceride metabolism: comparison with postheparin plasma. Metabolism.

[66] Watanabe H, Miyashita Y, Murano T (1999). Preheparin serum lipoprotein lipase mass level: the effects of age gender, and types of hyperlipidemias. Atherosclerosis.

[67] Totsuka M, Miyashita Y, Ito Y (2000). Enhancement of preheparin serum lipoprotein lipase mass by bezafibrate administration. Atherosclerosis.

[68] Seip RL, Mair K, Cole TG (1997). Induction of human skeletal muscle lipoprotein lipase gene expression by short-term exercise is transient. Am J Phys.

[69] Zhang JQ, Smith B, Langdon MM (2002). Changes in LPLa and reverse cholesterol transport variables during 24-h postexercise period. Am J Physiol Endocrinol Metab.

[70] Tofighi A, Rahmani F, Jamali Qarakhanlou B, Babaei S (2015). The effect of regular aerobic exercise on reverse cholesterol transport A1 and apo lipoprotein a-I gene expression in inactive women. Iran Red Crescent Med J.

[71] Butcher LR, Thomas A, Backx K (2008). Low-intensity exercise exerts beneficial effects on plasma lipids via PPARgamma. Med Sci Sports Exerc.

[72] Kamani CH, Gencer B, Montecucco F (2015). Stairs instead of elevators at the workplace decreases PCSK9 levels in a healthy population. Eur J Clin Investig.

[73] Rocco DD, Okuda LS, Pinto RS (2011). Aerobic exercise improves reverse cholesterol transport in cholesteryl ester transfer protein transgenic mice. Lipids.

